# Neural Correlates of Illusory Line Motion

**DOI:** 10.1371/journal.pone.0087595

**Published:** 2014-01-27

**Authors:** Jeff P. Hamm, Trevor J. Crawford, Helmut Nebl, Matthew Kean, Steven C. R. Williams, Ulrich Ettinger

**Affiliations:** 1 Cognitive Neuroscience Research Group, School of Psychology, The University of Auckland, Auckland, New Zealand; 2 Centres for Aging Research & Human Learning and Development, Department of Psychology, Lancaster University, Lancaster, United Kingdom; 3 Study Programme Psychology, University of Regensburg, Regensburg, Germany; 4 Manchester Medical School, Manchester, United Kingdom; 5 Centre for Neuroimaging Sciences, Institute of Psychiatry, King's College London, London, United Kingdom; 6 Department of Psychology, University of Bonn, Bonn, Germany; University of Rome, Italy

## Abstract

Illusory line motion (ILM) refers to a motion illusion in which a flash at one end of a bar prior to the bar's instantaneous presentation or removal results in the percept of motion. While some theories attribute the origin of ILM to attention or early perceptual mechanisms, others have proposed that ILM results from impletion mechanisms that reinterpret the static bar as one in motion. The current functional magnetic resonance imaging study examined participants while they made decisions about the direction of motion in which a bar appeared to be removed. Preceding the instantaneous removal of the bar with a flash at one end resulted in a motion percept away from the flash. If this flash and the bar's removal overlapped in time, it appeared that the bar was removed towards the flash (reverse ILM). Independent of the motion type, brain responses indicated activations in areas associated with motion (MT+), endogenous and exogenous attention (intraparietal sulcus, frontal eye fields, and ventral frontal cortex), and response selection (ACC). ILM was associated with lower percept scores and higher activations in ACC relative to real motion, but no differences in shape-selective areas emerged. This pattern of brain activation is consistent with the attentional gradient model or bottom-up accounts of ILM in preference to impletion.

## Introduction

The ability to detect motion is an evolutionarily old function that has been essential for the survival of both prey and predators. Indeed our sensitivity to movement is so ingrained that humans have a strong bias to perceive motion, even in the absence of any physical motion. The sensation of motion typically coincides with neural activations in specialised motion-sensitive areas of the visual cortex, most prominently the MT+ complex [1]. Imaging studies have shown that activation in MT+ is not limited to situations involving real motion, but can also be elicited by static images that induce the percept of motion, such as the Enigma illusion [2], the Rotating Snake illusion [2], [3], moving illusory contours [4], [5], or other types of apparent motion [6], [7], [8], [9], [10], [11], [12].

One motion illusion that has received considerable interest is referred to as illusory line motion (ILM). When a luminance flash precedes the sudden presentation or disappearance of a bar, the bar seems to be drawn in motion away from the location of the flash [13], [14]. This illusion of motion occurs if the flash and the bar are in relatively close spatial proximity of each other [15], [16], [17], [18]. Typically the cue and bar are adjacent, though some studies include small gaps and ILM continues to be obtained [17], [19], [20]. With large separations (>4.2°) the direction of the illusion may reverse [18], although this is not always found [20].

Explanations for ILM can broadly be divided into bottom-up and/or attentional gradient hypotheses [13], [14], [21], [22], [23] and a top-down hypothesis based on an inferential process, referred to as impletion [19], that is required to resolve an ambiguous signal. Attention based explanations generally attribute the resulting motion percept to the prior entry of visual signals that is triggered by the attention capturing properties of the flash [24]. The prior entry benefits are thought to be created by a gradient of attention [18], [25], [26], [27], [28] that is centred on the flash [29], and therefore the prior entry benefits are likewise distributed as a gradient. The notion is that because stimuli are detected more quickly when attended then the onset or offset of the section of the bar near the flash is detected earlier in time than the onsets/offsets at more distant sections. Because the prior entry benefits are thought to be distributed as a gradient the result is a gradient of perceptual onsets or offsets similar to those that occur when an onset bar or offset bar is actually in motion.

In contrast, impletion is based upon the idea that rapid inference is required (though this is below the level of conscious awareness) because the physical display is itself ambiguous in that it could represent either a line in motion or the less probable sudden appearance/disappearance of a visual stimulus. Given that objects do not usually suddenly appear or disappear out of the blue it is argued that the evolutionary history of the visual system has generated a bias against such an interpretation. If the idea of an improbable new object is discounted, this leaves open the more viable implicit inference that real motion must be present and this motion signal is then supplied by the process of impletion [19].

In another explanation of the impletion type ILM is regarded as a specific case of “transformational apparent motion” (TAM), a term for motion illusions with temporally segregated but spatially overlapping stimuli in which subjects perceive a change in shape in addition to a change in position [30]. TAM is thought to result from high-level mechanisms that detect different shapes or forms (“parsing”) and then match them across time to solve the correspondence problem, which eventually cumulates in the percept of motion. This hypothesis is supported by behavioural studies showing that a suddenly appearing bar flanked by two boxes seems to extend out of the box that has a similar luminance as the bar [31]. Cowan and Greenspahn [32] investigated apparent motion type displays using a paradigm where participants indicated when the motion reached a marker, either placed at the mid-point of the apparent motion path or at the end of the motion path. Their results indicated that response times for the mid-location were not faster than the response times to the end points, indicating that the motion was back-projected in time after the initial perception of the end point. Interestingly, a similar study employing ILM suggested that the motion in this illusion was not back-projected, at least under some conditions [33].

Interactions between form and motion pathways have also been postulated by Baloch and Grossberg [34]. In contrast to TAM they assume competition between orientation-selective bipole and hypercomplex cells located in early visual areas up to MT+ to be sufficient to result in a faster processing at one edge of the bar. According to their view, ILM should arise without any attentional or higher-order mechanisms involved, although both can modify the processing. Evidence comes from studies of standard apparent motion, in which continuous motion is perceived between two temporally and spatially separated stimuli. Muckli and colleagues [9] presented a square alternating from the upper to the lower part of one hemifield and compared periods of reported motion with periods of subjective blinking. MT+ was the only area of the visual cortex activated differentially, in accordance with results by Zhou and co-workers [12]. For ILM stimuli Jäncke and colleagues [35] demonstrated that a box followed by a bar induces a spatio-temporal activation profile in cat visual cortex similar to that of a moving square. The motion percept in ILM stimuli would thus result from propagating activations in early visual cortex, closely resembling a true motion signal. In a computational model proposed by that group, lateral interactions in V1 were sufficient to explain ILM [36]. ILM as a pre-attentive bottom-up phenomenon was further supported by a more comprehensive computational model of the lateral geniculate nucleus and the primary visual cortex [37]. The perceived motion in ILM stimuli was fully explained by the assumption of long-range lateral connections between neurons in V1, in the absence of any top-down feedback from higher cortical areas. These explanations of ILM, unlike impletion, suggest that the motion percept arises directly, due to early modification of the visual input such that the processed signal becomes equivalent to that generated by a line in motion. Thus the steps of specialised parsing/matching mechanisms terminating in a back-projected motion are eliminated.

In summary, bottom-up theories of ILM emphasize either the importance of lateral connections within early visual cortices [36] or suggest that the luminance flash acts as an exogenous cue to create an attentional gradient of prior entry benefits. In contrast, impletion or object-based accounts assume higher-order mechanisms either fully underlie ILM or they at least play a major role [19], [30].

While ILM has been investigated through behavioural paradigms, so far only one EEG study [38] and one preliminary fMRI study by Tanabe and Yanagida [39] have addressed the neural mechanisms of ILM. In the fMRI study [39] involving five participants, a circle served as a cue and was followed by a bar, which resulted in activations in MT+, lingual gyrus, parietal lobe, frontal eye fields and supplementary motor area. These findings are generally consistent with an attentionally-driven account, but are limited insofar as ILM was not compared to any control condition. Thus the engaged attentional networks might be a correlate of visuo-spatial processing per se. Activations in lingual gyrus might also correspond to form and shape selective areas of the lateral and ventral visual cortex [40], which would rather favour impletion theories.

To differentiate between these accounts the current fMRI study measured the blood oxygen level dependent response (BOLD) while observers viewed displays that produce ILM, real motion, and a flash-line condition that was not expected to result in any sensation of motion.

Similar to other types of motion illusions ILM should activate motion-sensitive area MT+. If ILM arises due to low-level processes recreating the input signal of a line actually in motion, activations in early visual cortices up to MT+ should be indistinguishable from those occurring in the presence of real motion. Signals in early visual cortices might also result from feedback of higher visual areas though, as suggested by Sterzer and colleagues [10] for apparent motion stimuli.

If ILM is due to a gradient of attention or a bottom-up mechanism not requiring specialised parsing/matching mechanisms, then activations are predicted to be restricted to motion (MT+) and both the endogenous and exogenous attention networks [41], and no further areas should be engaged during the ILM condition. In addition, if the ILM display results in an exogenous shift of attention then networks associated with orienting of attention should likewise be active, particularly the temporal-parietal junction (TPJ; [41], [42]), and the ventral prefrontal cortex [41]. Since subjects were required to report the direction of perceived motion of a stimulus with a very short duration, endogenous attention networks are likely to be activated independent of the condition. Such networks would correspond to frontal eye fields and areas along the intraparietal sulcus (IPS; [39], [41]). Thus the attention networks might be activated by unspecific task demands or the peripheral flash despite not playing a causal role in the perception of ILM.

According to impletion theory, attention itself is not sufficient to produce ILM but attention may bias the direction of impletion. Hence, if ILM arises as a result of impletion or other higher-order mechanisms then additional networks representing the “higher level processes that ensure object continuity and coherence” [19] should be evident when compared to real motion. One likely candidate region for impletion might be the ventral part of the lateral occipital complex (LOC), which was associated with increased activations during motion-inducing TAM stimuli compared to control stimuli [43]. The posterior parietal cortex could be another critical node of an impletion network. Based upon a single-cell study in monkeys this cortical area has been implicated to represent the higher-order filling-in process of apparent motion [44].

## Method

### Participants

Nineteen participants completed the study (9 males; 10 females; mean age  = 27.58 years, SD = 6.07, range  = 20–43). Participants were recruited amongst university staff and students. They were physically, neurologically, and psychiatrically healthy and denied consumption of any prescription or over-the-counter medication on the day of scanning. All were right-handed as assessed by the Edinburgh Handedness Inventory [45]. The study was approved by the Joint Institute of Psychiatry and Maudsley Hospital Research Ethics Committee and participants provided written informed consent before participation. After data collection had been completed one subject had to be excluded from the study due to scanner artefacts.

### fMRI Data Acquisition

Participants underwent fMRI at 1.5 Tesla on a SIGNA HDx scanner (General Electric, Milwaukee, Wisc., USA) equipped with an 8-channel headcoil for radiofrequency transmission and reception. T2*-weighted echo planar images of the whole head depicting the blood oxygen level dependent (BOLD) response were acquired yielding 720 volumes aligned parallel to the intercommissural plane (AC-PC line), each with 27 slices of 5 mm thickness and 0.5 mm gap. fMRI parameters were: repetition time (TR)  = 3000 ms, echo-time (TE)  = 40 ms, flip angle (FA)  = 90°, field of view  = 24 cm, NEX  = 1. The duration of the actual experiment, which was carried out in one run, was 36 minutes. Following the functional series a high-resolution T1-weighted anatomical axial gradient-spoiled, gradient recalled (SPGR) scan with an inversion time (TI) of 300 ms was acquired. The sequence was acquired with an isotropic resolution of 1.1×1.1×1.1 mm^3^, FA = 18°, TR = 4.84 ms and TE = 4.84 ms.

### fMRI Procedure and Task Design

Participants were placed supine in the scanner bore and viewed the screen via double mirrors. Stimuli were back-projected onto the screen. Participants held a button box in their right hand and an emergency button in their left hand and were connected to the control room via headphones and microphone. Their heads were stabilised in the headcoil using foam padding in order to minimise movement.

**Figure 1:**
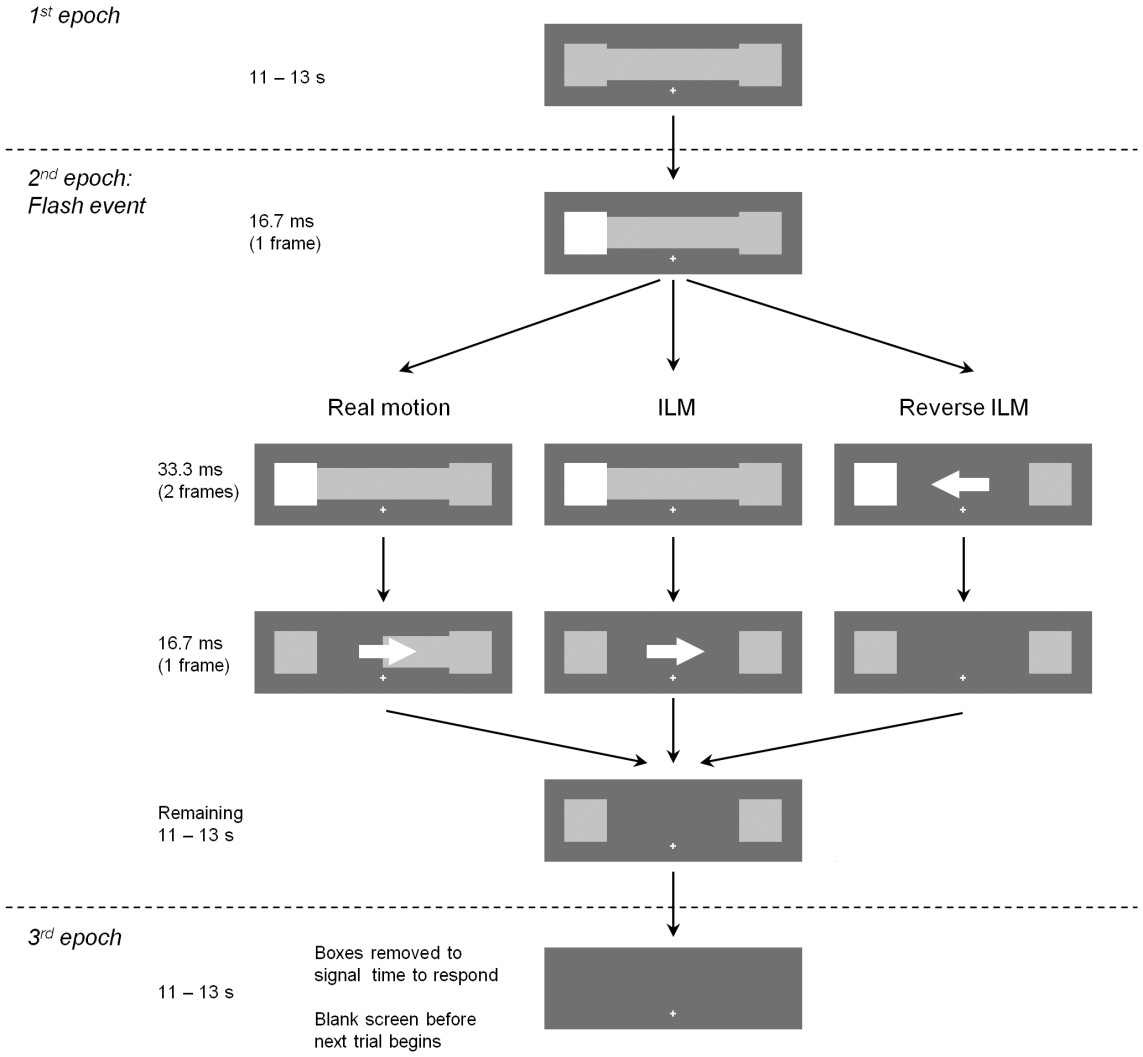
Diagram depicting a trial sequence for the real motion, ILM, and reverse ILM conditions.

The experiment employed an event-related design with three conditions, namely “illusory line motion” (ILM), “real motion”, and a condition that was intended as a “no motion control” but which will be referred to as “reverse illusory line motion” (reverse ILM) for reasons to be explained later. The trial structure was the same for each condition and consisted of three epochs, namely (1) the appearance of the horizontal bar and adjacent squares, (2) the event of interest (which differed between conditions, see below), and (3) the disappearance of the squares signalling the subject to respond. Each of these epochs lasted on average 12 seconds but durations were jittered between 11 and 13 seconds. Splitting up a single trial into these three epochs ensured that the event of interest, i.e. the percept of real or illusory motion, was not confounded by response execution in general and differences in reaction times in particular, which might result from easier decisions for stimulus material containing physical motion. There were 20 trials in each condition (10 left and 10 right flash trials), which were presented in the same quasi-random sequence for each participant. A diagram depicting the trial sequences is shown in figure 1, below.

In the first component of each trial the stimuli were displayed in the centre of the screen. They consisted of a horizontal bar (7.7° in width and 1.8° in height) in grey (value 180, on a greyscale from 0 - black, to 255 - white) presented on a dark grey background (value 90). On each side of the bar (right and left) was a square of the same colour (2.5° x 2.5°). A white fixation cross was presented throughout the trial sequence and was located below the centre of the bar. These stimuli remained on the screen for the first component of each trial with no requirements for participants to respond.

The second component of a trial, the event of interest, involved the turning off of the horizontal bar in three conditions, viz. either in one step after the flash of a square (ILM), in two steps after the flash of a square (real motion), or in one step during the flash of a square (reverse ILM).

In an ILM trial one of the two squares flashed (i.e. changed to white, value 255, for three screen refresh rates; 50 ms) and immediately following this the bar was turned off by changing its colour to equal the background - value 90. The stimulus onset asynchrony (SOA) of the square flashing and the bar disappearing was 50 ms. As described above, this sequence was expected to create the illusion of movement down the bar starting in the location of the square that flashed.

In a reverse ILM trial the bar disappeared during the flash. Specifically, the sequence was to increase the luminance of the box, value 255, wait a single screen refresh, remove the bar in its entirety on the second screen refresh, value 90, and wait a third screen before returning the box to its starting luminance, value 180, creating a 16.7 ms box flash – bar removal SOA. This sequence was not expected to cause a perception of motion [14], [21], [22] as the line was removed during the flash rather than at its offset. As presentation of the line prior to the cue produces ILM towards the cue, rather than away from the cue [27], it was predicted that removing the line during the flash should disrupt ILM, particularly if ILM arises due to impletion processes as this should create confusion between the option of impleting by combining the line's removal with the flash onset, which occurs before the line removal, or with the flash offset, which occurs after the line removal and reverses the direction of ILM [27]. However, because participants reported motion towards the flash this condition will be referred to as the reverse ILM condition.

In a real motion trial the square flashed and on the same frame as the flash offset (SOA = 50 ms) the bar disappeared in two steps, with each half being removed on successive screen refreshes, creating stroboscopic motion in the direction away from the square [15]. The flash was included to keep the physical stimuli comparable to the other two conditions. Despite the flash impletion should not occur in this situation because the real motion in the display is not an ambiguous signal.

In all conditions, following the disappearance of the bar the squares remained on the screen for the remaining duration of the second component of the trial. Participants were to withhold their response until the third component of the trial.

At the beginning of the third component of the trial the squares were turned off. This signalled to the participants to press a button on a keypad in order to indicate the direction of their perceived motion. Keypads were held in the right hand and had two buttons representing right and left. Participants were asked to respond on each and every trial and were instructed to guess if they were unsure. A left response was scored as -1 and a right response was scored as +1, providing a mean perceptual score between -1 for all trials perceived as left to +1 indicating all trials perceived as moving to the right. A score of 0 indicated no consistent direction of motion occurred. It should be noted that mean percept scores are a simple linear transformation of proportion of responses rightward and can be converted to such by simply adding one and dividing by two.

Following the turning off of the squares, no further stimuli were presented for the duration of the third epoch, and the next trial began immediately with the presentation of stimuli as described above.

### fMRI Data Analysis

Pre-processing and analysis of fMRI data were carried out with SPM8 (Wellcome Department of Imaging Neuroscience, London, UK) running under Matlab 7.5 (Mathworks, Natick, MA, USA). The origin of the acquired images was set to the anterior commissure according to definitions of the Talairach space. Serving as dummy-data-acquisition to allow for saturation of the magnetic field the first four functional volumes were excluded. Functional images were then slice-time corrected with the temporally middle slice serving as reference and realigned to the mean image of the time series. After coregistration the structural image was segmented and normalised into MNI standard space using unified segmentation [46]. The normalisation parameters were reapplied to the functional images, which were subsequently resampled to 2×2×2 mm^3^ voxel size and smoothed with an 8 mm full-width at half-maximum (FWHM) Gaussian kernel.

Data were analysed within the framework of the general linear model. At the single-subject level the implemented haemodynamic response function was convolved with stick functions (event-related) representing the onsets of the experimental conditions. Slow signal drifts and temporal correlations between the residual errors were removed employing a high-pass filter of 1/128 Hz and an auto-regressive AR(1) model.

The analysis of interest focussed on the second trial component. Trials with responses in the correct (for real motion) or expected direction (for the two illusion conditions) were modelled separately for the three conditions. While there were a total of 868 trials with responses in the correct or expected directions, nine of these were excluded from the fMRI analysis as the response was made during the second trial component and so would introduce artefacts due to motor activity. Trials with incorrect responses (real) or unexpected direction responses (ILM and reverse ILM) were collapsed across conditions and modelled by a fourth regressor. Two additional regressors accounted for changes of the visual input at the beginning of the first and third components, respectively. Realignment parameters were included to control for interpolation errors during the realignment procedure.

Single-subject level contrast images were generated for each of the three conditions and also for pair-wise comparisons. Resulting individual contrast images were entered into random effects analyses to investigate the underlying activation patterns at the group-level using one-sample t-tests.

The threshold of significance at the voxel level was set at *p*_corr_<0.05 (FWE) for first order contrasts and at an initial *p*_uncorr_<0.001 for second order contrasts. Clusters were considered as significantly activated when surpassing a minimum size of k = 20 voxels and a threshold of *p*_corr_<0.05 (FWE) at the cluster level. For clusters differing significantly between conditions the corresponding percentage signal change values were extracted with MarsBaR 0.42 [47].

Anatomical regions were identified using the Anatomical Automatic Labeling (AAL) toolbox [48]. For visualisation purposes the resulting activations were mapped onto the population-average landmark- and surface-based (PALS) standard brain [49] with Caret 5.6 [50]; http://brainvis.wustl.edu/wiki/index.php/Caret:About. Additional images were generated using MRIcron (http://www.mccauslandcenter.sc.edu/mricro/mricron/index.html).

## Results

### Behavioural Results

**Figure 2:**
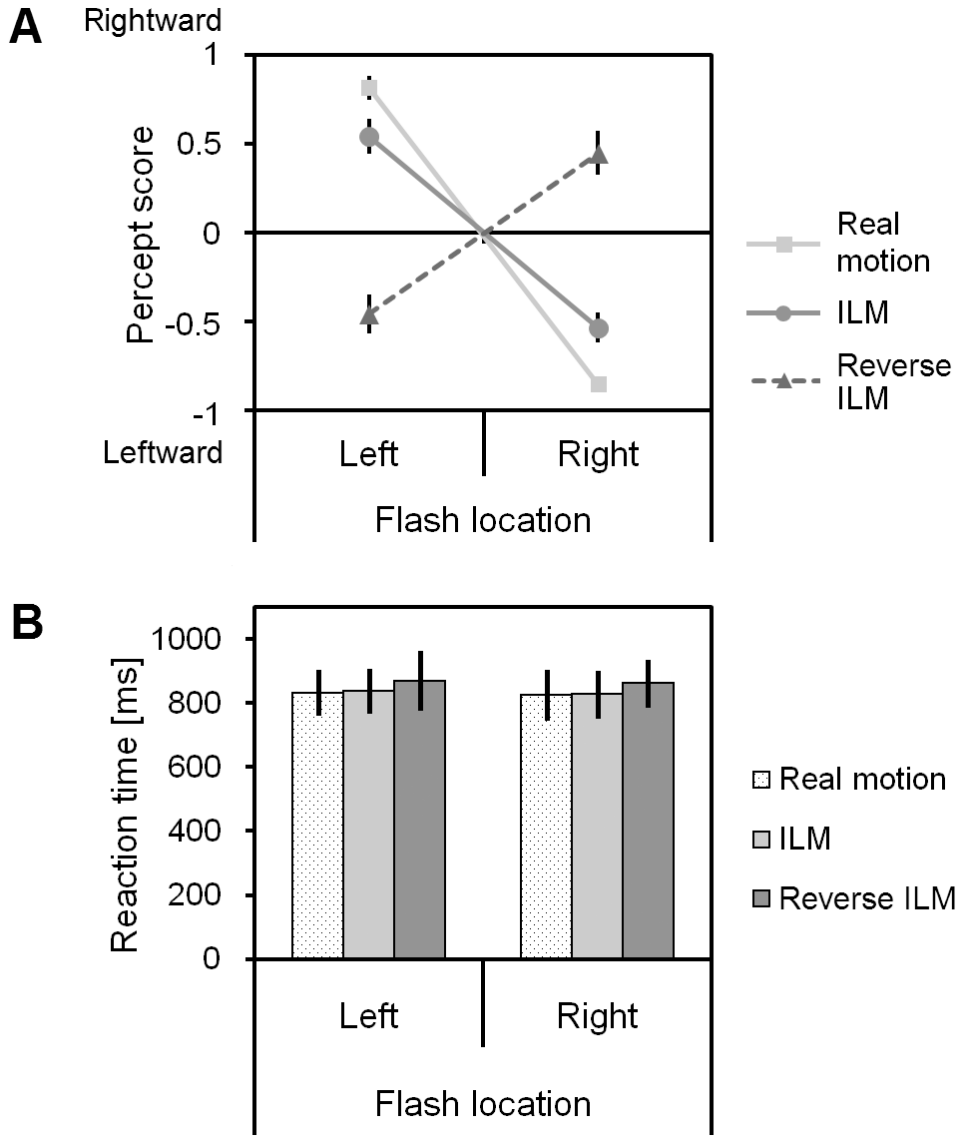
Behavioural data as a function of flash location and experimental condition. Panel A) shows Percept scores and B) Decision Times. Error bars indicate +/− 1 SEM. ILM: illusory line motion.

Percept scores were analysed in a two-way, within-subjects ANOVA with flash location (left/right) and condition (real, ILM, reverse ILM) as factors. This resulted in a main effect of flash location (*F*_(1,17)_ = 40.06, *p*<0.05) with left flashes producing more positive scores than right flashes (0.302 vs. −0.313 for left and right flash locations, respectively). There was no effect of condition (*F*_(2,34)_ = 0.06, *p*>0.05). The flash location by condition interaction was significant (*F*_(2,34)_ = 127.36, *p*<0.001) and is depicted in Figure 2. As can be seen, the intended “no motion control” condition resulted in motion towards the flash, rather than no motion, which is why we refer to this condition as “reverse ILM”. The interaction between flash and condition remained significant when the reverse ILM condition was dropped from the analysis (*F*_(1,17)_ = 20.60, *p*<0.05), indicating motion perception was more consistent in the real motion condition.

Considering motion away from the flash as the expected direction in the ILM and real motion conditions, and motion towards the flash as the expected direction in the reverse ILM condition, there were an average of 18.3, 15.4, and 14.5 trials responded to as in the expected direction for the real, ILM, and reverse ILM conditions, respectively. Wilcoxon signed ranks test was used to compare the ILM and reverse ILM conditions, and the number of trials responded to as being in the expected direction did not differ between conditions (*p*>0.5). These were averaged and compared to the number of trials in the real motion condition, and it was found there were more trials in the expected direction for the real motion condition (*p*<0.001).

Decision times were analysed in a similar manner. There were no significant main effects or interactions as a result of this analysis (all *p*>0.2, grand mean response time 843 ms).

### fMRI Results

**Figure 3:**
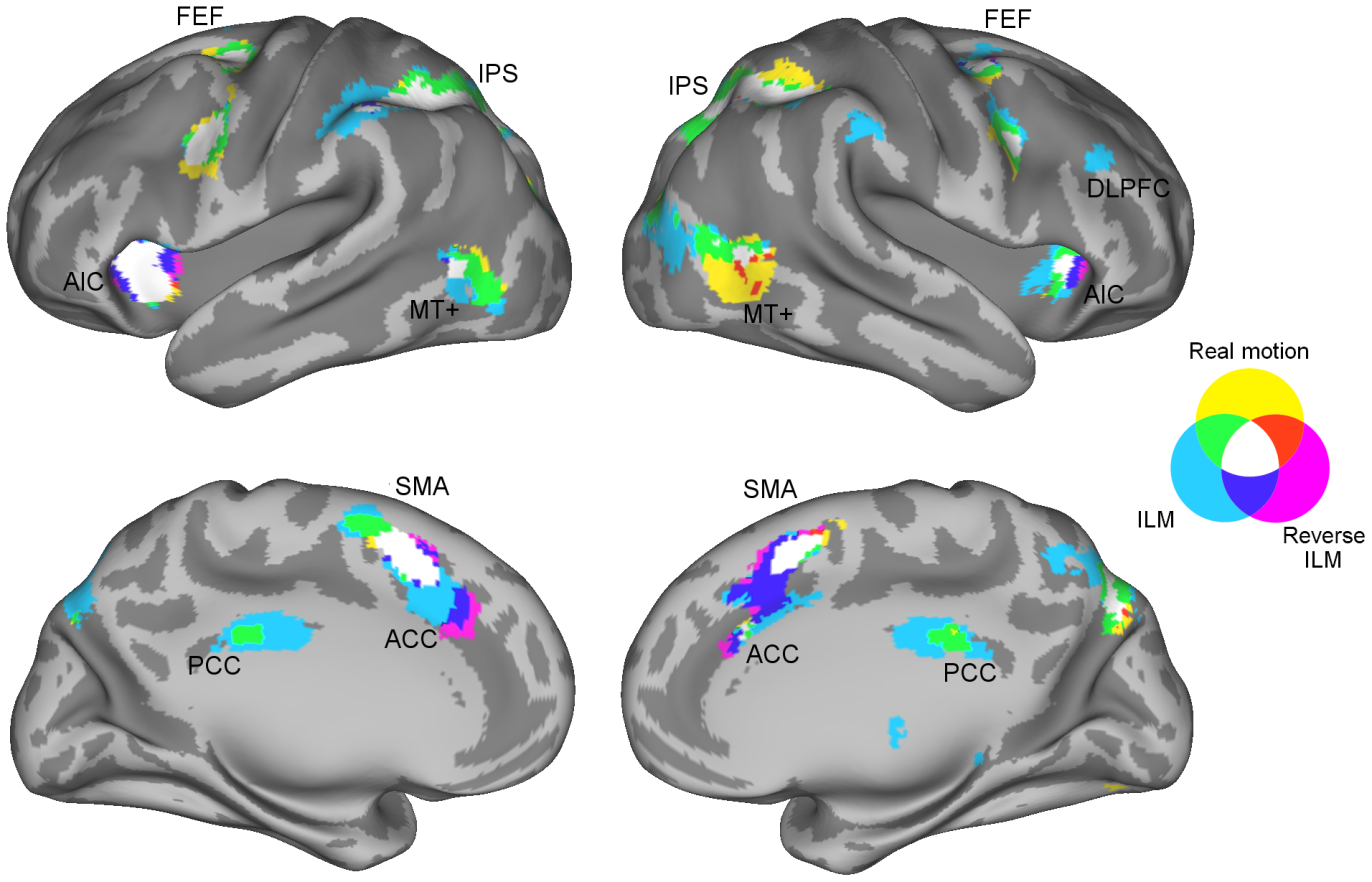
Activations for each of the three motion conditions superimposed onto the population-average landmark- and surface-based (PALS) standard brain (voxel threshold *p*_corr_<0.05 FWE, k>20). ACC: anterior cingulate cortex, AIC: anterior insular cortex, DLPFC: dorsolateral prefrontal cortex, FEF: frontal eye field, ILM: illusory line motion, IPS: intraparietal sulcus, PCC: posterior cingulate cortex, SMA: supplementary motor area.

During the second trial component (flashing of the box and disappearance of the bar), each of the three conditions was associated with significantly increased activations (Tables 1 – 3 and Figure 3). The engaged network included widespread occipito-parietal activations along the intraparietal sulcus extending into the precuneus and superior/middle occipital gyrus as well as activations in anterior insular cortex, middle frontal/precentral gyrus, supplementary motor area and anterior cingulate cortex (ACC). Bilateral activations in the middle temporal gyrus are in accordance with previously reported coordinates of MT+ complex (see discussion).

**Table 1:** Clusters of Activation in the Real Motion Condition (voxel threshold *p*_corr_ = .05 FWE).

			MNI Coordinates				
Brain Region	Side	Label	x	y	z	k	*T*
Inf. parietal, sup. parietal, middle occipital	L	IPS (incl. MT+)	−38	−52	50	1 818	15.40
Precentral, sup. frontal, middle frontal	L	FEF	−50	2	36	714	13.87
Middle temporal, inf. parietal, middle occipital, sup. occipital	R	(incl. MT+)	18	−72	46	2 644	13.60
SMA	L/R	SMA	−4	8	54	624	12.20
Precentral, frontal operculum	R	-	42	4	32	228	11.24
Insula	L	AIC	−32	22	6	298	10.84
MCC	L/R	-	−6	−30	28	94	9.39
Middle frontal, precentral	R	FEF	38	4	50	97	9.24
Insula	R	AIC	34	22	6	78	8.70
MCC, ACC	R/L	ACC	10	24	26	48	8.38
Fusiform gyrus	R	-	30	−72	−10	21	8.11

ACC: anterior cingulate cortex, FEF: frontal eye field, inf.: inferior, IPS: intraparietal sulcus, L: left, MCC: middle cingulate cortex, R: right, SMA: supplementary motor area, sup.: superior.

**Table 2:** Clusters of Activation in the Illusory Line Motion Condition (voxel threshold *p*_corr_ = .05 FWE).

			MNI Coordinates				
Brain Region	Side	Label	x	y	z	k	*T*
Precentral	L	-	−52	2	36	352	15.77
SMA, MCC	R/L	SMA (incl. FEF L)	8	20	36	1 875	14.59
Middle occipital, inf. parietal, precuneus	R	(incl. MT+)	22	−66	46	2 897	14.36
Inf. parietal, sup. parietal, middle occipital	L	IPS	−34	−58	48	2 419	14.17
MCC	R/L	-	4	−18	26	383	13.15
Insula	L	AIC	−34	20	2	747	13.11
Hippocampus, lingual gyrus, precuneus	R	-	20	−38	−2	36	10.53
Precentral, middle frontal, frontal operculum	R	FEF	40	2	30	498	10.43
Insula	R	AIC	36	14	−4	326	10.19
Middle temporal, middle occipital, inf. occipital	L	MT+	−50	−64	4	244	8.88
Pars triangularis, middle frontal	R	DLPFC	44	32	26	50	8.66
Thalamus	R	-	12	−8	8	31	7.48

For abbreviations see Table 1.

**Table 3:** Clusters of Activation in the Reverse Illusory Line Motion Condition (voxel threshold *p*_corr_ = .05 FWE).

			MNI Coordinates				
Brain Region	Side	Label	x	y	z	k	*T*
Insula	L	AIC	−38	14	0	708	15.60
Inf. parietal, angular gyrus, sup. occipital	R	IPS	38	−42	40	729	12.69
Inf. parietal, sup. parietal	L	IPS	−34	−52	46	1 146	12.39
SMA, MCC	R/L	SMA	4	16	50	1 022	11.71
Precentral	L	-	−44	0	30	277	10.84
Middle frontal, sup. frontal, precentral	L	FEF	−26	−4	52	112	10.05
Middle occipital	R	-	30	−72	22	130	9.20
Middle frontal, precentral	R	FEF	34	2	56	55	8.58
Insula	R	AIC	32	26	0	87	8.51
Middle temporal	L	MT+	−50	−64	4	21	8.26
Middle temporal	R	MT+	50	−54	6	111	8.24
Frontal operculum, precentral	R	-	42	8	30	21	8.10

For abbreviations see Table 1.

**Figure 4:**
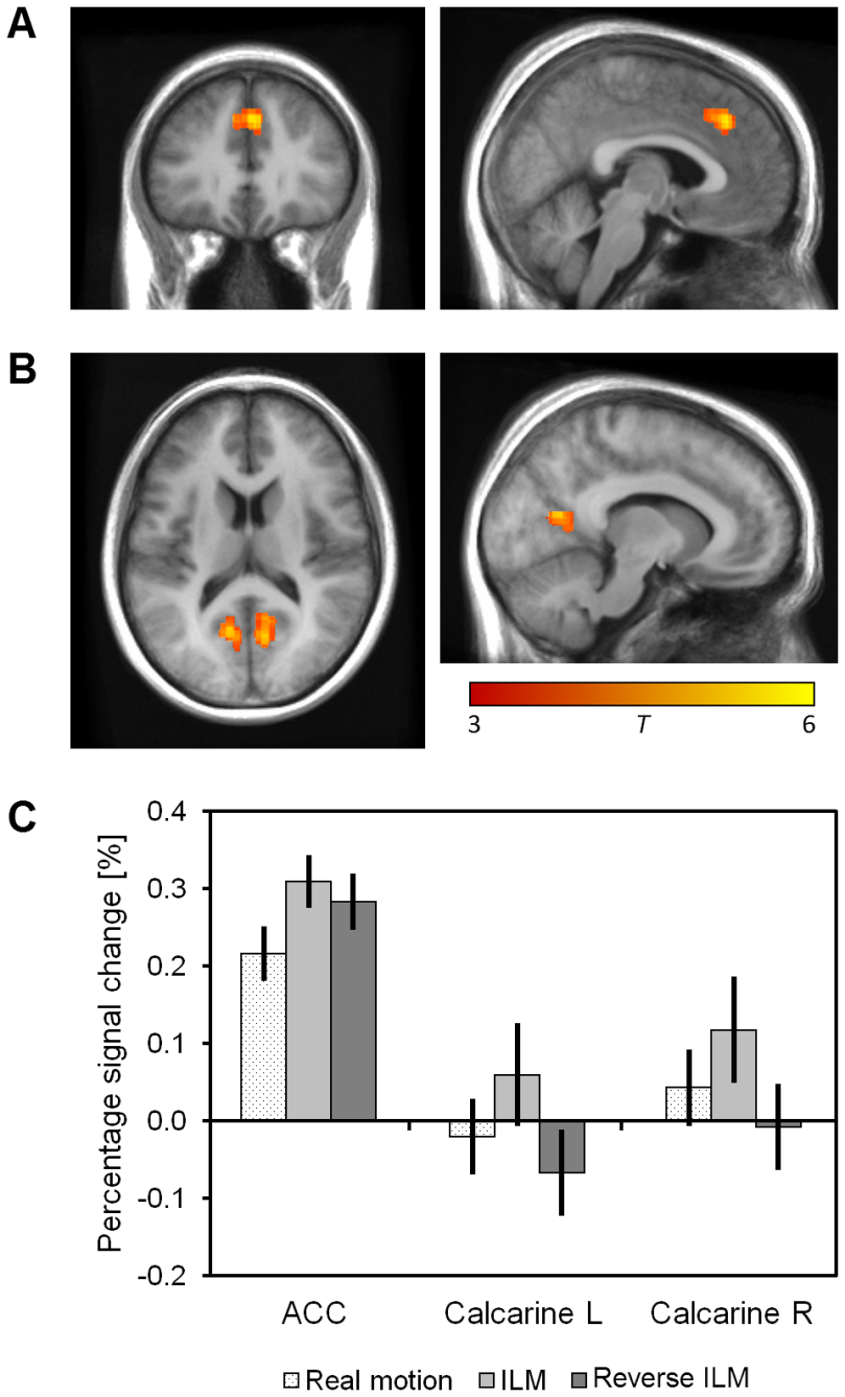
Differential brain activations (initial voxel threshold *p*_uncorr_<0.001, cluster threshold *p*_corr_<0.05 FWE) overlaid on the study-specific average of the normalised anatomical scans for the contrast “Illusory line motion > Real motion” (A), the contrast “Illusory line motion > Reverse illusory line motion” (B) and associated percentage signal change values (C). Error bars indicate +/− 1 SEM. ACC: anterior cingulate cortex, ILM: illusory line motion, L: left, R: right.

The differential contrast ILM> real motion yielded one statistically significant cluster located in the ACC and the medial part of the superior frontal gyrus (see Figure 4A and Table 4). For descriptive purpose we extracted percentage signal change values averaged across this cluster, showing that the activation level during reverse ILM was lying in between, but more closely to ILM (see Figure 4C).

**Table 4:** Differences in Activation between the Three Motion Conditions (initial voxel threshold *p*_uncorr_<0.001, cluster threshold *p*_corr_<0.05 FWE).

		MNI Coordinates				
Brain Region	Side	x	y	z	k	*T*
Illusory line motion > Real motion						
Superior frontal medial, ACC	L/R	4	32	36	371	5.37
Illusory line motion > Reverse illusory line motion						
Calcarine sulcus, lingual gyrus, precuneus	R	8	−56	16	273	5.13
Calcarine sulcus, precuneus	L	−10	−62	14	158	4.96

For abbreviations see Table 1.

ILM> reverse ILM yielded two significant clusters at the intersection of the calcarine and the parieto-occipital sulci of the right and the left hemisphere (see Figure 4B and Table 4). ILM was associated with slightly positive percentage signal change in contrast to reverse ILM with slight deactivations (see Figure 4C). However, the values did not differ significantly from zero in any of the three conditions, meaning there was no evidence of a significant modulation by the stimuli.

The other differential contrasts did not show any significant clusters. Additionally, there was no significant correlation between the activation in the ACC cluster and the difference in the number of correct responses during ILM and real motion trials when modelling ILM activity greater than real motion.

## Discussion

### Behavioural results

The behavioural results of our study indicated that participants had no difficulty in detecting the real motion in the direction away from the flashed box. When the bar was removed in its entirety on the same frame as the flash offset the participants also generally indicated that the bar was removed in the direction away from the flash; this finding confirms the illusory line motion (ILM) effect. The responses were more consistently in the direction away from the flash in the real motion condition than in the ILM condition, as indicated by the interaction in the percept scores between the flash location and condition when only the real and ILM conditions were compared. This is consistent with findings previously reported [51] including a study involving a similar display [15], and suggests that the motion in the real motion condition is more salient^1^ compared to the motion generated by ILM alone. Note, we do not equate salience with speed in this case. It seems likely that the relationship between the perceptual salience of motion and the speed of motion would best be described as an inverted U, with saliency dropping off for both very fast and very slow moving stimuli. While the current study only employed conditions where real motion and ILM could combine, in Crawford et al. [15] real motion in the absence of a flash was less salient than real motion with a flash, suggesting the ILM signal combined with the real motion signal to yield a net increase in perceived motion. It should be noted that as impletion is described as a process that serves the purpose of disambiguating ambiguous input, then the presence of real motion in the display should not require impletion. In other words, once there is an unambiguous motion signal there should be no evidence of impletion creating an additional ILM signal that combines with the real motion signal, especially in a manner that serves to increase the ambiguity by cancelling the perception of the real motion when the ILM would be in opposition [15]. An alternative line of reasoning that may partially fit with impletion is that while attention may not produce the illusory motion, attention may serve to enhance detection of the real motion, and so the improved performance is not due to the combination of signals from ILM and real motion. However, this explanation does not account for why the motion is less salient when ILM and real motion would be in opposite directions [15]. While beyond the scope of the current study, an in depth exploration of these combinations of real motion and ILM working in concert and opposition suggest a promising and interesting line of research. Finally, during the current experiment, if the bar was removed after the onset of the flash but before the offset of the flash, participants reported the bar as being in motion but towards the flash. This perception was of similar magnitude as the motion away from the bar in the ILM condition (as shown in Figure 2).

According to the attentional gradient theory for ILM the motion is perceived because the capture of attention by the flash results in faster detection of the line offsets near the flashed location. This runs counter to the suggestion that stimulus offsets are delayed by attention due to attention producing a temporal extension of the stimulus [27], meaning the section of the line near the flash should be perceived for a longer duration than the sections further away. However, some support for a temporal extension effect may be found in the reverse ILM condition, where the line is removed in the midst of the flash, or after the onset but before the offset of the flash. With the participants indicating that the line was removed as if disappearing under the flashed location, we assume that this condition has produced ILM in the opposite direction to that normally obtained and so we refer to this as reverse ILM. If attention underlies both the ILM and reverse ILM illusions, then there must be a short lived period of temporal extensions rapidly followed by prior entry benefits for detecting the line offset. These temporal extensions may correspond to the inhibitory process produced by peripheral cues that is normally masked by attentional cuing [52] in that the detection of the offsets near the flash may be briefly inhibited by the short cue lead time (16.7 ms) which turn into benefits by 50 ms in the ILM condition due to attentional facilitation. However, if remains unclear whether this inhibitory process, which Danziger and Kingstone [52] suggest is inhibition of return (IOR), produces delayed perception of visual stimuli or simply inhibition of responses to stimuli in that location [53], and so this interpretation must be considered as speculative. Note that standard impletion accounts cannot explain the perceived motion towards the flash for reverse ILM. Instead, one would expect motion towards the un-flashed box due to equivalent luminance [30], [31].

### Neural correlates

#### Early visual areas

The comparison of ILM with reverse ILM revealed significant clusters in the anterior part of the calcarine sulcus, corresponding to the far peripheral parts of the visual field [54], [55], which is beyond the range of standard fMRI set-ups [56]. Recently developed wide-field stimulations covering a field of view of up to 120° along the horizontal axis [57], [58] suggest that early visual areas V1 and V2 extend towards the parieto-occipital sulcus, as previously proposed by cytoarchitectonic studies [59], [60], [61]. With the current set-up, the stimuli were presented within the central 15° of the visual field, making in quite unlikely that the detected differences in activation were related to direct physical stimulation. Imperfect registration or inter-subject variability might have resulted in more anterior eccentricity representations of the stimuli in some of the subjects, but even if such inaccuracies occurred they cannot account for why conditions should differ. It has to be stressed that the peripheral stimulation (stationary box on the one side and flashing box on the other side) was equal in all three conditions.

As the two illusion conditions did not differ from real motion in these areas the outcome is difficult to interpret. Following the observation, ILM and reverse ILM seem to result in slightly different modulations of the periphery. Interestingly, Muckli and colleagues [8] observed coactivations more peripheral to the cortical representations of their stimuli for both apparent motion and real motion. They assume that top-down feedback results in filled-in activations not only along the motion path, but also peripheral to the stimuli due to large receptive fields of neurons in higher visual cortices. Even so, differences between conditions in the periphery might also have emerged from bottom-up spreading activations.

#### MT+ complex

The real motion condition resulted in robust bilateral activations near the lateral intersection of the occipital and temporal lobe, in contrast to the first event even at a more liberal threshold (data not presented). Similarly located motion sensitive areas of the lateral occipital cortex, summarized as MT+ complex, have previously been identified by PET and fMRI studies using flicker stimuli, moving dot patterns, or retinotopic mapping procedures [1], [62], [63], [64].

Both ILM and reverse ILM also were associated with activations in the MT+ complex, with descriptively smaller clusters in the reverse ILM condition compared to ILM and real motion. This might point to a less consistent motion signal arising from the reverse ILM condition since the BOLD signal in MT, a subregion of MT+, increases with a more consistent motion signal [65]. Furthermore, a direct relationship between the strength of perceived motion and the activation level in MT has been demonstrated in the case of motion aftereffects [66], [67]. However, when contrasting the three conditions no differences emerged, indicating that the apparent reduction in cluster size for the reverse ILM condition is not considered reliable. It is acknowledged that the lack of a no motion condition, with corresponding lack of activation in the MT+ area, makes it impossible to assert with total confidence that the activity observed in the region of MT+ is reflective of motion perception and not, for example, reflective of some higher-order impletion process. However, the most parsimonious explanation is that the activity represents motion perception that is thought to have occurred in all three of the present conditions based on the behavioural data.

#### Other higher visual areas

The relevance of occipital regions more ventral to MT+ have previously been emphasised in the context of TAM perception and could therefore reflect an impletion network. Contrasting TAM displays with control stimuli that did not evoke any percept of motion Tse [43] observed increased activations in ventral aspects of the LOC including the posterior fusiform gyrus. These findings might correspond to activations of the lingual gyrus reported in Tanabe and Yanagida [39] 's investigation of ILM. However, in the present data no activations were evident in the ventral aspects of the visual cortex during any of the motion events, nor were object-selective cortices modulated differently by real motion compared to illusory motion. Therefore, if the activation in LOC reflects impletion during TAM perception then the current data shows no evidence for the involvement of impletion during ILM. This difference between TAM and ILM on the neural level would be consistent with the different patterns found in similar behavioural paradigms (compared to [32], [33]).

Different outcomes might partly be attributed to specific settings of previous studies. In contrast to Tanabe and Yanagida [39] and Tse [43], the present design employed three different epochs allowing the direct targeting of the motion event. Although the percept of motion is vivid, it lasts for a short period only. Tse [43] might have introduced a bias by collapsing periods of motion alternating with periods of no-motion within a “motion” block. It remains unclear whether differences in LOC between these TAM sequences and control sequences represent activations due to parsing/matching mechanisms or due to rapidly changing sensations (from the possible mixing of alternate motion and no-motion periods) during TAM compared to the more uniform control sequence that would be more prone to adaption over time. Besides, the activation profile was heightened already at the level of V1 and successive early visual areas, as confirmed by additional ROI analyses conducted in a subset of subjects, possibly reflecting a top-down modulation. As TAM compared to the no-motion control condition also revealed differences in insular cortex, which has been linked to attention (for reviews see [68], [69], [70]), it cannot be excluded that commonly increased activations in visual areas during TAM were due to (unspecific) attentional modulation rather than due to specific scene processing mechanisms.

In conclusion, the results do not provide any evidence for an involvement of higher-level impletion type processes for scene or object perception during ILM, but are compatible with attentional type or related theories assuming a perceptually driven “spreading activation” in early visual areas.

#### Attention networks

The overall pattern of the BOLD signal during the three conditions of interest revealed several regions linked to endogenous and exogenous attention [41], [68]. Pronounced activations were located in the IPS, frontal eye fields, and ventral frontal cortex, although no activations were found in the temporo-parietal junction. The involvement of both attentional networks in response to ILM has previously been suggested based upon ERP results [38]. It should be noted that the two systems are not considered as distinct as originally presented [68].

In the current study, additional activations were evident in the anterior insular cortex, which has been proposed to coordinate sensory networks and to play an integral role for saliency detection and task control together with the anterior cingulate cortex [69]. Activations were also present in the precuneus, which is connected to both the inferior and superior parietal lobules, anterior cingulate, and frontal areas including the frontal eye fields, and appears activated in various attention related tasks (see [71] for a review).

None of the reported areas differed between real motion and the illusion conditions. Following attentional accounts, attention should be captured by the flash, eventually leading to the impression of motion during ILM. As the flash was an integral part of the stimulus material in all three conditions, exogenous attention networks would be activated to the same extent.

#### Cingulate and frontal cortices

There were, however, differences between ILM and the real motion condition in areas other than the two attentional networks and the motion area MT+. Such areas require further consideration as possible evidence for an impletion process. First, the ILM condition showed increased activity in ACC relative to the real motion condition. The ACC has been reported to reflect competition between stimuli [72] and decision conflicts [73], [74]. Decision conflicts may result from the ILM condition generating a less consistent or less salient motion signal relative to the real motion condition, which would place the signal closer to a response decision boundary and that results in an increase in decision conflict [75]. In other words because the real motion condition combined ILM with physical motion this would result in a more salient motion signal, placing it further from the response decision boundary, which would in turn reduce the response conflict and therefore lead to lower ACC activity. This coincides with the behavioural data, as the percept scores were indeed lower for the ILM condition when compared with real motion. With ILM being weaker in those diagnosed with schizophrenia [15], who also show thinner ACC grey matter [76], this finding warrants further investigation. It is relevant to note, however, that Pardo et al. [73] have implicated the ACC as part of the attention network, which would mean this activation may not be outside of the attention network after all.

Although not significantly different from either, the ACC activity in the reverse ILM condition was closer to that of the ILM condition than to that of the real motion condition, a trend which would be compatible with the idea that this activity reflects a conflict arising due to the reduced saliency of the overall motion signal relative to that of the real motion condition [75]. Still, the trend for lower activations during reverse ILM relative to ILM would imply a less severe decision conflict. It may be that the ILM condition contains an additional conflict in response selection between responding to the direction of the motion signal and a tendency to respond towards the location of the flash [16]. Because the reverse ILM condition would place both signals, the flash location and the motion of the line, in a compatible relationship this might reduce the decision conflict somewhat. However, as the comparison between ILM and reverse ILM was not significant this suggestion is presented for future considerations should this general pattern replicate and prove to be a reliable finding. For the present study, these suggestions simply serve as examples that interpretations for this activation other than impletion are readily available from the literature and so this activity cannot be taken as evidence for impletion.

Second, the ILM condition was associated with activity in the dorsolateral prefrontal cortex (DLPFC) that did not arise in the real motion or reverse ILM condition. However, in direct comparison there was no support for differences between conditions, indicating that interpreting the DLPFC as an impletion area is unwarranted. Activity in the DLPFC may simply reflect the maintaining of an attentional set to respond to the direction of the motion, or some other such task demand, since the execution of the response was to be delayed until well after the presentation of the stimuli [41].

### Conclusions

In summary, areas of activation implicate both the endogenous and exogenous attentional networks to be engaged during displays that produce ILM. Similar areas appeared when the display contained real line motion. All three conditions showed activity in MT+, and while the absence of a no motion control condition weakens the strength of this conclusion, it is consistent with the suggestion that the behavioural patterns do reflect decisions based upon motion perception. Illusory and real motion displays differed only in areas associated with response selection (ACC), which probably corresponds to the lower consistency in responding in the ILM condition. An intermediate activation profile in the ACC for the reverse ILM condition suggests that response conflict may have been lower relative to ILM, which would be consistent with a reduced conflict from the location of the flash. Finally, there was no indication of activations other than those associated with attention, motion, and response selection, which is in agreement with the attentional gradient model for ILM or bottom-up accounts that assume ILM to arise due to low-level attention or perceptual mechanisms in early stages of the visual system. The lack of activations in shape- or object-selective areas like the LOC runs counter to impletion theories for ILM, which propose that an ambiguous signal has to be processed within specialised networks and only then results in the percept of motion. The findings suggest that our bias to perceive motion may, in part, be a result of attentional processes, which could also feedback to influence earlier visual processing regions.
